# Commentary on “Nutritional screening and extraglandular manifestations in Sjögren disease: a cross sectional study using the controlling nutritional status score and prognostic nutritional index”

**DOI:** 10.1186/s42358-026-00589-1

**Published:** 2026-11-02

**Authors:** Dong Zheyu, Shi Jieyi

**Affiliations:** Hangzhou TCM Hospital Affiliated to Zhejiang Chinese Medical University (Hangzhou Hospital of Traditional Chinese Medicine), HangZhou, Zhejiang 310007 China

We sincerely appreciate the valuable study by Ösken et al. [1], which innovatively combined the CONUT and PNI indices to explore the relationship between nutritional status and extraglandular manifestations (EGM) in patients with Sjögren’s syndrome. Nevertheless, we would like to put forward several constructive comments to further improve this excellent work.

First, we noted a logical inconsistency in the cutoff definition of PNI and potential multicollinearity bias in the multivariate regression model. The study adopted the formula PNI = (10 × serum albumin [g/dL]) + (0.005 × total lymphocyte count [/mm³]), with higher PNI values reflecting better nutritional status. Consistent with this definition, the original study reported that patients with EGM had significantly lower PNI levels than those without EGM (49.3 vs. 54.5). However, the ROC analysis in the same study defined the predictive cutoff as PNI > 51.625 for EGM prediction. Based on the above results, a more reasonable cutoff should be revised to PNI ≤ 51.625, whereby lower PNI values indicate a higher risk of EGM occurrence. In addition, the multivariate regression model simultaneously incorporated ESSDAI, CONUT, and PNI as independent variables, which may induce significant multicollinearity. Notably, both CONUT and PNI are calculated based on serum albumin and peripheral lymphocyte counts, and the ESSDAI score also contains hematological parameters. The high overlap in variable composition will inevitably lead to strong internal correlation, resulting in unstable odds ratio values and biased statistical results. We therefore recommend retaining only one nutritional index (either CONUT or PNI) to optimize the reliability and robustness of the regression model.

Second, the inherent limitation of cross-sectional design and unadjusted confounding factors may affect the credibility of the causal inference. As a cross-sectional study, it is difficult to clarify the causal direction between malnutrition and EGM, namely whether malnutrition contributes to the development of EGM or EGM-related systemic inflammation leads to subsequent malnutrition. More importantly, the study failed to collect and adjust for immunosuppressive treatment data. Glucocorticoids, commonly used in the clinical management of Sjögren’s syndrome, can significantly alter nutritional and hematological indicators by inducing hyperglycemia, reducing serum albumin levels, and redistributing peripheral lymphocytes [2]. The lack of adjustment for glucocorticoid exposure and dosage may confound the true association between nutritional status and EGM, thereby introducing unavoidable selection bias.

Third, the dual inflammatory-nutritional properties of CONUT and PNI limit the ability to distinguish different types of malnutrition. CONUT and PNI are composite prognostic markers that reflect both systemic inflammation and nutritional status [3]. Relying solely on these two indicators cannot effectively distinguish protein-energy malnutrition from inflammation-induced catabolism. Comprehensive collection of dynamic BMI changes and quantitative dysphagia severity data would help differentiate the two pathological processes and further elaborate the underlying mechanism of nutritional disorders in Sjögren’s syndrome patients with EGM.

In conclusion, we highly commend the authors for their innovative and rigorous research. We hope our modest suggestions will help refine the statistical analysis and conclusion of this study, and we look forward to more high-quality researches from the authors in this field.

## Data Availability

No datasets were generated or analysed during the current study.
